# Factors Influencing Burnout in Croatian Medical Students: The roles of Lifelong Learning and Loneliness

**DOI:** 10.5334/pme.1468

**Published:** 2025-05-13

**Authors:** Ivan P. Gradiski, Ana Borovecki, Marko Curkovic, Esperanza García-Gómez, Roberto C. Delgado Bolton, Luis Vivanco

**Affiliations:** 1 Specialist Psychiatric Office Ivan Pavao Gradiski MD, 10000 Zagreb, Croatia; 2 Faculty of Medicine, University of Zagreb, 10000 Zagreb, Croatia; 3 Department of Statistics, Mathematics and Informatics, Miguel Hernández University of Elche, 03202 Elche, Spain; 4 Cantabrian Health Service, 39011 Santander, Spain; 5 Platform of Bioethics and Medical Education, Center for Biomedical Research of La Rioja (CIBIR), Logrono, Spain; 6 Faculty of Health Sciences, International University of La Rioja (UNIR), Logrono, Spain

## Abstract

**Background:**

Burnout and engagement are seen as opposite ends of a continuum. In medical education, engagement reflects motivation and social belonging, while burnout signifies a lack of interest in learning and social detachment. This study aimed to investigate the influence of lifelong learning and loneliness on these dynamics.

**Methods:**

A cross-sectional study involving a culture back-translation procedure was performed. Participants were medical students enrolled in a Croatian medical school. The Maslach Burnout Inventory (MBI-GS), Jefferson Scale of Physician lifelong learning (JeffSPLL-MS), and the Scale of Social and Emotional Loneliness Scale for Adults (SELSA-S), were used as main measures. Sex, age, grade point average, and year of study, were collected in a complementary form. Confirmatory Factor Analyses (CFAs), followed by correlation, comparative and multiple linear regression analyses were performed to assess the above-mentioned variables.

**Results:**

The sample consisted of 1,371 medical students (872 women), all native Croatian speakers. A model including lifelong learning, loneliness and sex variables accounted for 17% of the variance in the global MBI-GS score. This model showed a medium-to-large effect size and fulfilled conditions required for statistical inference. Additionally, differences by sex appeared in loneliness (p < 0.001), but not in lifelong learning abilities. Furthermore, the Croatian versions of the JeffSPLL-MS and the SELSA-S exhibited good psychometric properties, as confirmed by CFAs.

**Conclusions:**

These findings highlight the influence of lifelong learning abilities and loneliness on the burnout-engagement continuum. Additionally, findings indicate that female medical students are at heightened risk of experiencing burnout.

## Introduction

### Background

Unlike workplace-related burnout, learning burnout refers to a combination of emotional exhaustion, cynicism towards one’s studies, and feelings of incompetence as a student [1,2,3,4]. It is estimated that almost half of all medical students will experience this type of burnout either during their training or professional careers [5], with the risk that it may lead to severe mental health problems such as psychiatric disorders or even suicidal ideation [6]. Engagement, as the opposite of burnout [7], is described as a positive, fulfilling, and enduring work-related state characterized by three attributes: vigor, dedication, and absorption [4,7]. In the context of medical education, vigor is associated with the energy invested in educational activities and the willingness and ability to put effort into academic tasks; dedication pertains to the sense of significance, enthusiasm, inspiration, and challenge that medical students feel in their training and in medicine as a professional goal; and absorption is associated with a state of involvement and enjoyment that medical students show in their learning activities. According to Leiter and Maslach [8], in addition to the extreme profiles of burnout and engagement, three additional profiles can emerge: disengaged (characterized by high cynicism only), overextended (marked by high exhaustion only), and ineffective (defined by inefficacy only).

Given that medical learning is a progressive and continuous process that begins in medical school and extends throughout a medical career [9], it is plausible that the aforementioned profiles may arise depending on the interplay between individual characteristics and environmental circumstances [10,11]. Equipping medical students with lifelong learning abilities from the earliest stages of their training may foster ongoing learning engagement and reduce the risk of burnout [11]. Lifelong learning abilities refer to a set of self-initiated activities (behavioral aspects) and information-seeking skills (capabilities), driven by sustained motivation (predisposition) to learn and an aptitude for recognizing one’s educational needs (cognitive aspects) [12]. Research evidence suggests that possessing these abilities is beneficial in preventing burnout among both medical students [11] and physicians [10,13]. However, medical learning encompasses more than individual abilities. Social interactions among trainees, trainers, and training institutions significantly influence learning goals and the risk of burnout. Social resilience, also called collegiality, and sense of belonging are two key aspects of how social interactions shape these outcomes [14]. In medical education, collegiality refers to students’ ability to handle stress positively through mutual trust and bonding. A sense of belonging, on the other hand, arises when medical students feel valued, accepted, and integrated within the medical community. A lack of social acceptance and support serves as a barrier to these conditions, increasing the risk of loneliness and social isolation among medical students. Loneliness –a multidimensional construct resulting from a deficit in qualitatively different relationships [15]– is a common issue among both medical students and physicians. It has been associated with dropout intentions and burnout in medical students [11,16,17,18], as well as among healthcare professionals [19,20] across various cultural contexts.

In addition to the role that learning abilities and loneliness play on the burnout-engagement continuum, other individual and environmental aspects are reported as influential. Among individual factors, having positive personal attributes –such as optimism, empathy, or grit– is negatively correlated with burnout [21,22]. Conversely, environmental factors such as studying in a competitive environment, experiencing social pressure, facing excessive workloads, or being in advanced medical courses are associated with a higher risk of learning burnout [2,23,24,25]. On the other hand, students who perceive support from their family and social environment demonstrate a reduced risk of burnout [11,24,26]. However, findings related to burnout differences based on academic performance and sex remain inconsistent. While some studies have reported a negative correlation between students’ burnout and grade point averages [27], others have found no relationship between burnout and grade and cumulative point averages [4,14,28]. Similarly, research comparing sex groups has shown mixed findings, with some studies reporting no association [29] and others indicating a higher risk of burnout in either female [24,28,30,31] or male [25,32] medical student groups. Some authors suggest that these inconsistencies may stem from various factors, including cultural differences, sample characteristics, and the measurements used [4,8].

In Croatia, undergraduate medical programs follow a six-year integrated curriculum that combines theoretical knowledge with practical training, in alignment with EU standards. The first three years, known as the pre-clinical phase, focus on theoretical knowledge and laboratory work. During this phase, students participate in a mix of large and small group learning activities facilitating social interactions primarily among trainees and some trainers. The last three years, referred to as the clinical phase, involve rotations in hospitals and healthcare settings, conducted in small groups. Learning activities during this phase are predominantly small-group-based, with rotations across different clinical environments. This allows students to interact with various trainers, clinical staff, and even patients. Throughout the program, students are continuously evaluated through exams, practical evaluations, participation in clinical activities, and a final exam. The entire process aims to enhance students’ learning abilities, collegiality, and sense of belonging. However, due to the need for constant adaptation to diverse learning demands in a hierarchical culture [33], it is plausible that medical students –especially in advanced courses– find their studies increasingly challenging over time if they do not develop adequate coping strategies.

### Study purpose

This study aimed to investigate the relationship between burnout and engagement in Croatian medical studies, focusing on two key elements: lifelong learning abilities and loneliness. It was hypothesized that medical students with poor learning engagement –characterized by a low score in lifelong learning abilities and a high perception of loneliness– report higher scores on measures of burnout. Four research objectives were established to validate this hypothesis: (i) To assess burnout, loneliness, and lifelong learning abilities in a sample of Croatian medical students; (ii) To measure the correlation between burnout, lifelong learning, and loneliness; (iii) To characterize differences in burnout based on socio-demographic variables (sex and age) and academic variables (grade point average and year of studies); and (iv) To analyze and quantify the relationship between burnout (as a dependent variable) and all variables that showed significant differences in previous analyses, while accounting for the effects of other variables.

## Methods

### Participants

Participants in this study were undergraduate medical students from the Faculty of Medicine at the University of Zagreb, Croatia. During the academic year 2018/2019, period when this study was performed, the entire population of undergraduate medical students enrolled in the Croatian medical program at this faculty was 1,969 students (1,231 female). Given that this faculty offers two medical programs, one in Croatian and another in English, only students enrolled in the program in the Croatian language were invited to participate. Medical students attending the program in English or enrolled in short internship programs (i.e. Erasmus) were excluded.

### Procedures

This study incorporated three scales: one for assessing burnout (MBI-GS) and two for measuring lifelong learning abilities (JeffSPLL-MS) and loneliness (SELSA-S), respectively. A validated Croatian version of the MBI-GS was provided by the authors of the instrument. However, the JeffSPLL-MS and SELSA-S scales required translation into Croatian, followed by adaptation and evaluation using a cross-cultural back-translation procedure [34]. This process was undertaken after obtaining written permissions from the respective authors, Dr. Mohammadreza Hojat and Dr. Enrico DiTommaso. Upon completion, the three scales, along with a socio-demographic form, were compiled into a single questionnaire, which was administered to all undergraduate medical students enrolled in the Croatian medical program during the 2018/2019 academic year. Questionnaires consisted of paper forms provided together with a pencil and an information letter in enclosed envelopes. Once students had responded to the questionnaires were responded they were returned in their envelopes to local researchers following a protocol previously approved by an independent ethics committee (Ref. 380-59-10106-17-100/159). The university provided administrative support for the process of distribution and collection of questionnaires. The participation was confidential, voluntary, anonymous and secret. There was no potential risk for participants, and participants could leave the study at any time.

### Psychometric measures

#### Burnout

The 16-item General Survey of the Maslach Burnout Inventory (MBI-GS) was used for measuring burnout [35]. The MBI-GS was initially designed with the main purpose of being administered to university students and occupational groups without direct personal contact with service recipients or with only casual contact with people [4,7,36]. This premise makes the MBI-GS the most suitable scale for the measurement of burnout in medical students, who usually have limited and always-supervised contact with the patients. Items of the MBI-GS are answered using a Likert Type scale from 0 (never) to 6 (always). The MBI-GS has three domains: “exhaustion”, “cynicism”, and “professional efficacy”. The “exhaustion” domain refers to both emotional and physical fatigue but does not make direct reference to people as the source of those feelings. The “cynicism” domain reflects indifference or a distant attitude towards the activity performed. Finally, the “professional efficacy” domain encompasses both social and non-social aspects of occupational accomplishments; this domain explicitly assesses an individual’s expectations of continued effectiveness at work. Together, the three domains of the MBI-GS provide a three-dimensional measure of burnout. According to the authors of the MBI [8], a “burnout” profile is characterized by high scores in the exhaustion and cynicism domains, combined with low scores in professional efficacy. A “disengaged” profile is observed when only high scores in cynicism are present; an “overextended” profile is identified when only high scores in exhaustion are reported; an “ineffective” profile is noted when only low scores in professional efficacy appear; and an “engagement” profile is identified by low scores in both exhaustion and cynicism, combined with high scores in professional efficacy.

#### Lifelong learning abilities

The 14-item Jefferson Scale of Physicians Lifelong Learning (JeffSPLL-MS) was used for measuring lifelong learning abilities [37]. These abilities refer to a set of skills related to information gathering, the use of learning opportunities, and the self-motivation in medicine. Items of the JeffSPLL-MS are answered using a Likert scale from 1 (strongly disagree) to 4 (strongly agree). A higher score indicates a greater development of lifelong learning abilities. The JeffSPLL-MS includes three domains: “motivation” (defined by learning beliefs and motivations), “attention” (described as attention to learning opportunities), and “skills” (referring to having technical skills in seeking information). A Croatian version of the JeffSPLL-MS was developed as part of this study.

#### Loneliness

The 15-item Social and Emotional Loneliness Scale for Adults (SELSA-S) was used for measuring loneliness in three specific environments: family, romantic relationships, and social contexts [38]. Loneliness is defined as the perception that one lacks meaningful connections with others, indicating an absence of interpersonal skills that is reflected in unsatisfactory human connections. The SELSA-S offers four possible measures: one global, and one by each specific environment. Items of the SELSA-S are answered using a Likert scale from 1 (strongly disagree) to 7 (strongly agree). A higher score indicates a greater perception of loneliness. A Croatian version of the SELSA-S was also developed as part of this study.

### Other measures

Additional data gathered included demographic information, with students providing the details in a supplementary form: sex, age, country of birth (Croatia or other), residential status (living in the same town as their family or not), year of studies, and grade point average (GPA) obtained in their last course (possible range: 0.00 to 5.00).

### Data analysis

Only the scales with fully completed scores in all their items were included in the statistical analysis. The reliability of each scale used was assessed by calculating Cronbach’s alpha coefficient (values higher than 0.70 were considered satisfactory). In the case of the JeffSPLL-MS and the SELSA-S, two independent confirmatory factor analyses (CFAs) were performed prior to any further analysis to discover whether the observed data fit the original postulated models. In contrast with a principal components analysis (PCA), the CFA is a theory-testing model that begins with an hypothesis prior to the analysis [39]. This hypothesis can be based on: (i) theory, (ii) previous research findings, or (iii) both [40]. In the current cases, both CFAs were based on the third case (theory and structure experimentally described by the developers of the JeffSPLL-MS and the SELSA-S, respectively). In both cases, a model based on a 3-factor structure was tested. Following the recommendation of other authors [41], a large sample size (with at least one thousand cases), with data non-normally distributed, and ordinal observed variables, was analysed using a robust weighted least squares (WLS) estimation method. On this basis, in each CFA analysis a robust WLS estimation method was used for the calculation of the structural equation modelling with ordinal observed variables with non-normality extremes Asymmetry >> 3 and Kurtosis > 8 [42,43]. As part of these analyses, the following parameters were estimated: goodness of fit indexes calculated to assess each model’s fit were χ^2^ statistics and its subsequent ratio with degrees of freedom (χ^2^/*df*), Comparative Fit Index (CFI), Tucker-Lewis Index (TLI), Root Mean Square Error of Approximation (RMSEA), and Standardized Root Mean Square Residual (SRMR). Models fit interpretation was based on the following criteria [41,44]: CFI and TLI values between 0.97 and 1.00 were considered as a good fit, and between 0.95 and below 0.97 were considered as an adequate fit; RMSEA values equal or smaller than 0.05 were considered as a good fit, and above 0.05 to 0.08 (included) as an adequate fit; finally, SRMR values below 0.05 were considered as a good fit, and above 0.05 to 0.10 (included) as an adequate fit.

Since none of the scales used followed a normal distribution, in comparative analyses by categorical variables, non-parametric tests (Mann Whitney U test) were used, while Spearman correlation coefficients were used to estimate the existence of association between burnout measure and the scales of lifelong learning, and loneliness. Spearman correlation coefficients were also calculated to assess associations between burnout and numerical variables.

In a further analysis, a model based on a multiple linear regression analysis was created using burnout as a dependent variable, while all variables with statistical significance in previous analyses were treated as possible explanatory variables. The model obtained was accepted once it met the following conditions: normality, zero mean, constant variance and independence of the residuals, in addition to linearity and absence of multi-collinearity. Finally, the degree of practical significance of this model measured by the effect size (Cohen’s *f^2^*), was calculated. An effect size equal to 0.02 was interpreted as small, equal to 0.15 was interpreted as medium, and equal to 0.35 was interpreted as large [45]. All these analyses were done in R Studio IDE (version 2023.06.1, for Windows).

## Results

The study sample included 1,371 medical students (response rate 70%), and 872 (64%) were women. Except for 12 (1%) students, who were born in Bosnia and Herzegovina, all participants were born in Croatia. In addition, all participants (including Bosnians) indicated they were Croatian native speakers. Average age was 21 years old (SD = 2; range 17–33). According to their living situation, 648 (48%) respondents were living in the same town as their families. According to their academic achievements, 275 (20%) students were enrolled in the first course, 270 (20%) in the second, 179 (13%) in the third, 250 (18%) in the fourth, 205 (15%) in the fifth, and 186 (14%) in the sixth course. Regarding students’ future medical interest, 457 (34%) indicated that they still had not decided which medical specialty to pursue in the future. Finally, the mean GPA was 3.90 (*SD* = 0.60), with a range between 0.70 and 5.00.

### Burnout, loneliness, and lifelong learning measures

**Figure 1:**
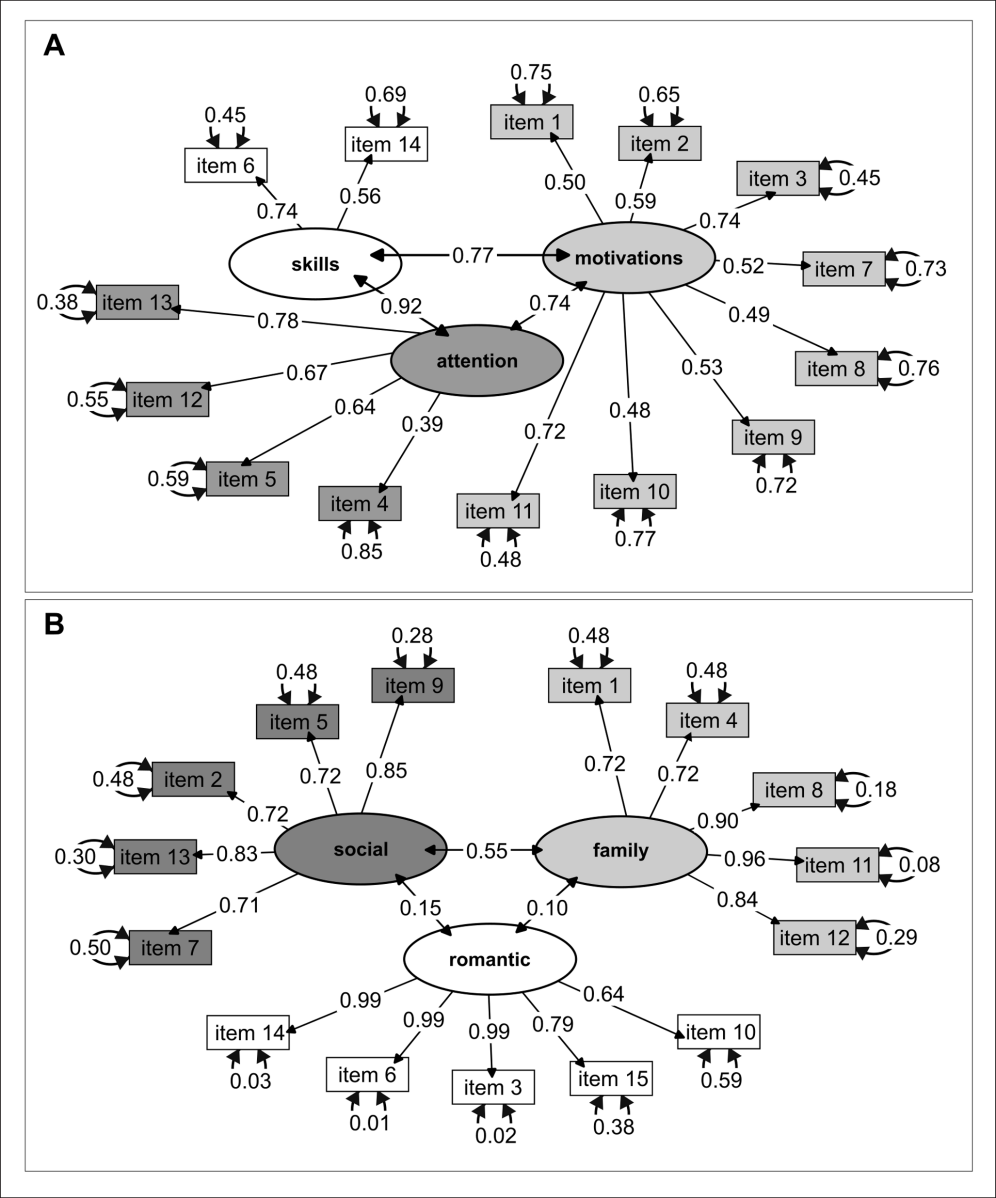
Models obtained from the Confirmatory Factor Analyses of the 3-factor structure of: **(A)** the Medical Student Version of the Jefferson Scale of Physicians Lifelong Learning (JeffSPLL-MS), including “motivations”, “attention”, and “skills” domains; and **(B)** the Social and Emotional Loneliness Scale for Adults (SELSA-S), including the domains of “family”, “romantic”, and “social” loneliness.

**Figure 2:**
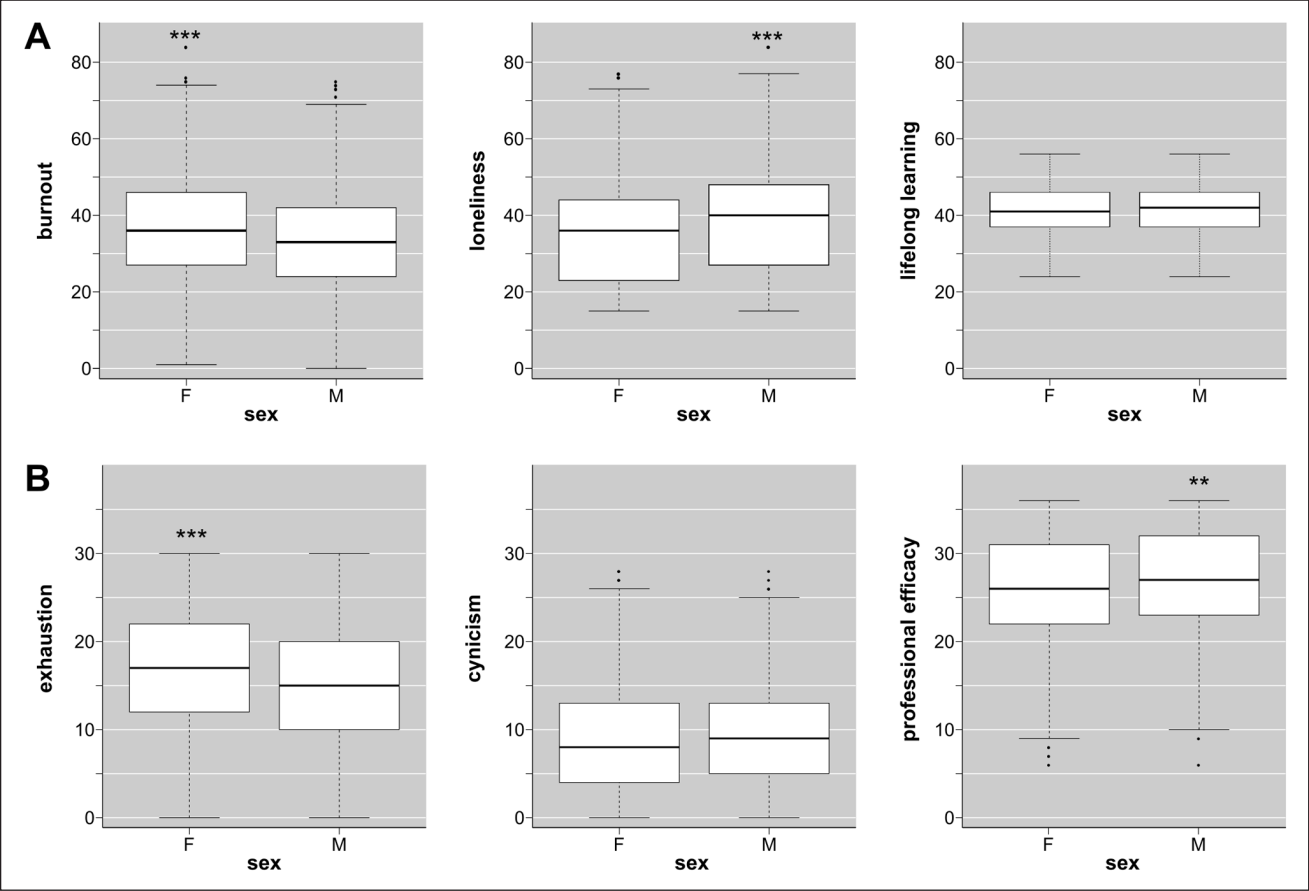
Comparisons between female (F) and male (M) medical students according to: **(A)** global score of “burnout” (MBI-GS), “loneliness” (SELSA-S), and “lifelong learning” (JeffSPLL-MS); and **(B)** “exhaustion”, “cynicism”, and “professional efficacy” domains. MBI-GS, Maslach Burnout Inventory – General Survey; JeffSPLL-MS, Jefferson Scale of Physicians Lifelong Learning – Medical student version; SELSA-S, Social and Emotional Loneliness Scale for Adults; ** *p* < 0.01; ***p < 0.001.

The first research objective was to measure burnout, loneliness, and lifelong learning abilities. All scales showed adequate reliability with Cronbach’s alpha coefficients higher than 0.70. Cronbach’s alpha coefficients were also higher than 0.70 by domains of the loneliness and burnout scales, but not by domains of the lifelong learning scale. Based on this preliminary finding, only global scores were used in further analyses. A summary of descriptive analyses for each scale and its domains is shown in the **Supplementary File 1**. In addition, a validation analysis was performed for the translated versions of the JeffSPLL-MS and the SELSA-S. Separate confirmatory factor analyses in the entire sample revealed an adequate data fit for the correlated 3-factor model of the JeffSPLL-MS and for 3-factor model of the SELSA-S. The 3-factor structure models obtained from these analyses for both scales are reported in Figure 1. Comparative analyses revealed differences by sex in loneliness (*p* < 0.001; *r* = 0.13), but not in lifelong learning (*p* = 0.76), as is shown in Figure 2.A. On this basis, three CFAs on the SELSA-S were conducted: one on the entire sample and two on sex groups. These analyses also revealed an adequate data fit for the correlated 3-factor model of the SELSA-S in the entire sample and on sex groups without representative differences. A summary of these analyses is shown in the **Supplementary File 2**. A printable version of the Croatian version of the SELSA-S is available in **Supplementary File 3**. For reasons pertaining to copyrights and permissions, the Croatian version of the JeffSPLL-MS is available upon request.

### Burnout is positively correlated with loneliness and negatively correlated with lifelong learning

The second research objective was to measure the correlation between burnout, lifelong learning, and loneliness. Burnout was positively correlated with loneliness (rho = +0.23; *p <* 0.001) and negatively correlated with lifelong learning (rho = –0.33; *p <* 0.001). In addition, a negative correlation was also confirmed between lifelong learning and loneliness (rho = –0.06; p = 0.02). A detailed report of these findings is presented in **Supplementary File 4**.

### Differences in burnout by socio-demographic and academic variables

The third research objective was to characterize differences in burnout based on socio-demographic (sex and age) and academic variables (grade point average and year of study). Regarding socio-demographic variables, Mann Whitney U tests confirmed differences by sex in overall burnout (*p* = 0.001; *r* = 0.09), exhaustion (*p* < 0.001, *r* = 0.14), and professional efficacy (*p* = 0.003; *r* = 0.08) domains, but not in cynicism (*p* = 0.07; *r* = 0.05). Additionally, two comparative analyses were conducted to examine differences in loneliness and lifelong learning measures by sex. These analyses confirmed differences in loneliness (*p* < 0.001; *r* = 0.13), but not in lifelong learning abilities (*p* = 0.76; *r* = 0.008). The effect sizes of all observed differences were small (*r* ≈ 0.10). A summary of these findings is shown in Figure 2. On the other hand, age was positively correlated with burnout (rho = +0.06; *p* = 0.043) with a small effect size (rho ≈ 0.10). Regarding academic variables, burnout was also positively correlated with year of studies (rho = +0.06; *p* = 0.043) with a small effect size (rho ≈ 0.10), but not with GPA scores (rho = +0.04; *p* = 0.25). Age and year of study generally align, with older students typically in advanced years. This assumption was validated through an additional correlation analysis (rho = +0.93; *p* < 0.001). A detailed report of all correlation analyses is provided in **Supplementary File 4**.

### Variables influencing the variability of medical students’ learning burnout

The fourth research objective was to identify factors influencing the variability of students’ burnout. A multiple linear regression analysis using burnout as a dependent variable allowed the creation of a model explaining 17.5% of the variability of the global score of the MBI-GS (*R^2^-*adjusted = 0.17; *F*_(3;1,199)_ = 84.76; *p <* 0.001; Cohen’s *f^2^* = 0.21) based on the following variables: “lifelong learning”, with a negative linear relationship, “loneliness”, with a positive linear relationship; and “being male”, with a negative linear relationship (Table 1). This model complied with all the conditions necessary for statistical inference. In addition, separate linear regression analyses were performed for each of the three domains of burnout. However, none of the models obtained fulfilled all conditions for statistical inference.

**Table 1:** Multiple linear regression models for burnout (MBI-GS).


EXPLANATORY VARIABLES	*β*	*SE*	*T*	*P*

*Global burnout*				

Lifelong learning abilities (JeffSPLL-MS)	–0.79	0.07	–12.90	<0.001

Self-perception of loneliness (SELSA-S)	+0.22	0.03	+7.97	<0.001

Sex (male students)	–3.30	0.78	–4.24	0.03


*Notes: β:* beta coefficient; *SE*: standard error; *t*: t experimental; *p*: p-value; MBI-GS: Maslach Burnout Inventory; JeffSPLL-MS: Jefferson Scale of Physicians Lifelong Learning; SELSA-S: Social and Emotional Loneliness Scale for Adults.

## Discussion

### Role of influence of lifelong learning and loneliness

This study aimed to investigate the influence of lifelong learning abilities and loneliness on learning burnout. It was assumed that lifelong learning abilities and meaningful relationships are beneficial for preventing burnout. Findings from a linear regression analysis confirmed this, indicating that lifelong learning acts as a protective factor, while loneliness serves as a risk factor, influencing students’ burnout. Regarding lifelong learning abilities, the findings align with the limited literature produced on this area [11,46,47,48]. On the contrary, the lack of meaningful relationships, represented by loneliness, appears as detrimental for the medical students’ well-being, making them more vulnerable to suffering burnout. This negative effect, directly derived from the lack of establishing adequate human connections that can bring emotional support, has been studied in different cultural and professional contexts [18,49], warning about the negative impact that loneliness has in individuals who are more exposed to be socially isolated due to their working duties [19,20]. However, the joint relationship between loneliness and learning with burnout has been scarcely studied. In this context, the findings reported in this study provide novel insights into how both aspects influence the burnout-engagement continuum within medical education contexts [4,14,50].

### Differences in burnout by sex

Findings observed in this study reveal sex-based differences in burnout among medical students. Female students report greater overall burnout compared to their male peers. Domain-specific comparisons also show higher emotional exhaustion in the female group, which may be associated with an “overextended” profile. Conversely, male students exhibit greater professional efficacy in comparison to their female peers. These findings are in consonance with previous studies reported in Serbia [28], Brazil [31], Pakistan [30], and Spain [24], reinforcing the notion that this may be a cross-cultural phenomenon. Different aspects may explain these differences, including disparities in self-perception of the academic achievement, emotional reasoning, stressful coping strategies, and the prevalence of certain professional sex roles within medicine.

### Burnout and academic achievement

Preliminary analyses confirmed that overall burnout is positively correlated with the year of study, but not with PGA scores. The correlation between burnout and the year of study is consistent with previous research [24,25], suggesting that as medical students advance in their training, the increasing demands and stressors contribute to a greater risk of burnout. The absence of a correlation between burnout and PGA scores is consistent with findings from other studies [3,14], which also reported no association between these variables. However, the positive correlation observed between PGA scores and cynicism highlights an aspect that requires special attention. Given the highly competitive academic environment in medical schools, it is plausible that achieving good grades not only offers better academic opportunities, but also professional advantages in the future. Nevertheless, prioritizing grades over other essential values in medicine, such as empathy, humanism, or altruism—core components of medical professionalism—can pose challenges. In this regard, the positive correlation between cynicism and PGA scores suggests the existence of an underlying problem when medical students fail to recognize this hierarchy of professional values.

### New tools for Croatian medical educators

The target group of this study were medical students enrolled in the Croatian medical program offered by the top-ranked Faculty of Medicine of Croatia. The authors predicted that only a minority of the participants were not born abroad but with Croatian family roots. This assumption was confirmed. Due to the nature of this work, lifelong learning and loneliness measures used had to be initially translated into the Croatian language. Since there was a previously validated Croatian version of the MBI-GS, this procedure was no longer necessary for this tool. As part of this procedure, CFAs confirmed the stability of the most relevant characteristics of the SELSA-S and the JeffSPLL-MS, as well as the existence of a 3-factor model structure in both cases.

### Limitations and strengths

This study may have three main limitations: (i) It is based on a cross-sectional design, which limits the ability to establish cause-effect relationships, while a longitudinal design would be required for such assessments. (ii) The findings explain only a small portion of the variability in learning burnout. Given its complexity, a comprehensive assessment of this phenomenon requires the analysis of additional factors beyond the scope of this study. (iii) The data were collected before the pandemic, providing a perspective unaffected by the confounding factors introduced by COVID-19. On the other hand, the strengths of this study include: (i) A large sample size. (ii) The use of scales with strong psychometric properties validated across different cultural settings. (iii) To the best of our knowledge, it is one of the few studies confirming a relationship among burnout, lifelong learning, and loneliness. Nevertheless, the findings from this study provide valuable insights for medical educators aiming to design interventions that enhance students’ mental health and academic performance.

## Conclusions

The main findings observed indicate that lifelong learning plays a protective role in the prevention of burnout, while loneliness is making students more vulnerable to suffering burnout. Sex-based differences in burnout have also been found. Overall burnout is positively correlated with age and year of study, but not with grade point average. Interestingly, grade point average demonstrates a positive correlation with cynicism, emphasizing the need to cultivate a stronger appreciation for the core values of medicine, which can sometimes be eclipsed by academic priorities.

All these findings could help having a better understanding of some aspects not directly associated with formal curriculum, that have an important impact on medical students’ burnout. These findings suggest that this phenomenon must not be overlooked. Medical educators and medical schools should be directed towards helping these students reduce the high prevalence of learning burnout.

To minimize and prevent burnout, the next steps should focus on implementing interventions that promote attributes of medical professionalism and foster a positive social environment. These measures should be introduced from the earliest stages of training and subsequently evaluated to assess their effectiveness.

## Data Accessibility Statement

Data are available upon reasonable request.

## Additional Files

The additional files for this article can be found as follows:

10.5334/pme.1468.s1 Supplementary File 1. Presenting descriptive analysis of scales of burnout, loneliness, and lifelong learning abilities.

10.5334/pme.1468.s2 Supplementary File 2. Presenting Goodness of fit indexes for the 3-factor models of the Croatian version of the JeffSPLL-MS and the SELSA-S.

10.5334/pme.1468.s3 Supplementary Table 3. Presenting the Croatian version of the SELSA-S.

10.5334/pme.1468.s4 Supplementary File 4. Presenting the Spearman correlation coefficients for the MBI-GS according to lifelong learning, point grade average, year of study, and age.

## Abbreviations

IG: Ivan P. Gradiski; AB: Ana Borovecki; MC: Marko Curkovic; EGG: Esperanza García Gómez; RDB: Roberto C. Delgado Bolton; LV: Luis Vivanco.
