# Supplementary material for: Factors Influencing Burnout in Croatian Medical Students: The roles of Lifelong Learning and Loneliness
Source: Perspect Med Educ. 2025 May 13;14(1):274–85. doi: 10.5334/pme.1468 (PMC12082462; doi:10.5334/pme.1468)
Supplement: Supplementary File 1. — Presenting descriptive analysis of scales of burnout, loneliness, and lifelong learning abilities. [file pme-14-1-1468-s1.pdf]

**Supplementary File 1.** Descriptive analysis of burnout, loneliness, and lifelong learning abilities.

| **Scales** | ***n*** | **PR** | **AR** | **M (*SD*)** | **Mdn** | **Reliability** |
|---|---|---|---|---|---|---|
| *MBI-GS* | 1,281 | 0-96 | 0-84 | 35.5 (14) | 35 | 0.84 |
| Exhaustion | 1,298 | 0-30 | 0-30 | 16 (7) | 16 | 0.87 |
| Cynicism | 1,291 | 0-30 | 0-28 | 9 (6) | 8 | 0.74 |
| Professional Efficacy | 1,287 | 0-36 | 6-36 | 26 (6) | 27 | 0.80 |
| *JeffSPLL-MS* | 1,347 | 14-56 | 24-56 | 41 (6) | 41 | 0.80 |
| Learning beliefs & motivation | 1,354 | 8-32 | 16-32 | 27 (3) | 27 | 0.67 |
| Attention to learning opportunities | 1,364 | 4-16 | 4-16 | 10 (3) | 10 | 0.62 |
| Skills in seeking information | 1,366 | 2-8 | 2-8 | 5 (1) | 5 | 0.51 |
| *SELSA-S* | 1,313 | 15-105 | 15-84 | 36 (14) | 37 | 0.81 |
| Family domain | 1,360 | 5-35 | 5-27 | 8 (4) | 6 | 0.82 |
| Romantic domain | 1,325 | 5-35 | 5-35 | 19 (11) | 23 | 0.90 |
| Social domain | 1,355 | 5-35 | 5-26 | 9 (4) | 8 | 0.80 |

*Notes:* MBI-GS: Maslach Burnout Inventory–General Survey; JeffSPLL-MS: Jefferson Scale of Physicians Lifelong Learning–Medical student version; SELSA-S: Social and Emotional Loneliness Scale for Adults; *n*: sample analysed; PR: possible range; AR: actual range; M: mean; SD: standard deviation; Mdn: median.
