# Supplementary material for: Factors Influencing Burnout in Croatian Medical Students: The roles of Lifelong Learning and Loneliness
Source: Perspect Med Educ. 2025 May 13;14(1):274–85. doi: 10.5334/pme.1468 (PMC12082462; doi:10.5334/pme.1468)
Supplement: Supplementary File 2. — Presenting Goodness of fit indexes for the 3-factor models of the Croatian version of the JeffSPLL-MS and the SELSA-S. [file pme-14-1-1468-s2.pdf]

Supplementary File 2. Goodness of fit indexes for the 3-factor models of the Croatian version of the Jefferson Scale of Physicians Lifelong learning for medical students (JeffSPLL-MS) and the Social and Emotional Loneliness Scale for Adults (SELSA-S) in the entire sample and by sex.

**Scale**	**X^2**	***df***	**Ratio X^2/*df***	**CFI**	**TLI**	**RMSEA**	**SRMR**
JeffSPLL-MS							
Entire sample	672.01	74	9.08	0.95	0.94	0.08	0.09
SELSA-S							
Entire sample	500.49	87	5.75	1.00	1.99	0.06	0.06
Male students	300.82	87	3.46	1.00	1.00	0.07	0.07
Female students	260.54	87	2.99	1.00	1.00	0.05	0.05

Notes: X^2: Chi-square statistics; *df:* degrees of freedom; CFI: comparative fit index; TLI: Tucker-Lewis index; RMSEA: root mean square error of approximation; SRMR: standardized root mean square residual
