# Supplementary material for: Factors Influencing Burnout in Croatian Medical Students: The roles of Lifelong Learning and Loneliness
Source: Perspect Med Educ. 2025 May 13;14(1):274–85. doi: 10.5334/pme.1468 (PMC12082462; doi:10.5334/pme.1468)
Supplement: Supplementary Table 3. — Presenting the Croatian version of the SELSA-S. [file pme-14-1-1468-s3.pdf]

Supplementary File 3. Croatian version of the Social and Emotional Loneliness Scale for Adults (SELSA-S)

Skala socijalne i emocionalne usamljenosti (SELSA-S)

Upute: Molimo označite u kojoj mjeri se slažete sa svakom od slijedećih tvrdnji.

Uopće se ne slažem						Potpuno se slažem
**1**	**2**	**3**	**4**	**5**	**6**	**7**

Tvrdnja							
1. Osjećam se sam kad sam sa svojom obitelji	**1**	**2**	**3**	**4**	**5**	**6**	**7**
2. Osjećam se dijelom skupine prijatelja	**1**	**2**	**3**	**4**	**5**	**6**	**7**
3. Imam romantičnog partnera/icu s kojim/kojom dijelim svoje najintimnije misli i osjećaje	**1**	**2**	**3**	**4**	**5**	**6**	**7**
4. Ne postoji nitko u mojoj obitelji na koga se mogu osloniti za podršku i ohrabrenje, ali bih volio da postoji.	**1**	**2**	**3**	**4**	**5**	**6**	**7**
5. Moji prijatelji razumiju moje motive i rasuđivanje.	**1**	**2**	**3**	**4**	**5**	**6**	**7**
6. Imam romantičnog ili bračnog partnera koji mi daje potporu i ohrabrenje koje mi je potrebno.	**1**	**2**	**3**	**4**	**5**	**6**	**7**
7. Nemam prijatelje koji dijele moje stavove, ali bi ih želio imati.	**1**	**2**	**3**	**4**	**5**	**6**	**7**
8. Osjećam se blizak svojoj obitelji.	**1**	**2**	**3**	**4**	**5**	**6**	**7**
9. Mogu se osloniti na svoje prijatelje za pomoć.	**1**	**2**	**3**	**4**	**5**	**6**	**7**
10. Želio bih imati ugodniju romantičnu vezu.	**1**	**2**	**3**	**4**	**5**	**6**	**7**
11. Osjećam se dijelom svoje obitelji.	**1**	**2**	**3**	**4**	**5**	**6**	**7**
12. Mojoj obitelji je uistinu stalo do mene.	**1**	**2**	**3**	**4**	**5**	**6**	**7**
13. Nemam niti jednog prijatelja koji me razumije, ali bih ga htio imati.	**1**	**2**	**3**	**4**	**5**	**6**	**7**
14. Imam romantičnog partnera čijoj sreći pridonosim.	**1**	**2**	**3**	**4**	**5**	**6**	**7**
15. Imam nezadovoljenu potrebu za bliskom romantičnom vezom.	**1**	**2**	**3**	**4**	**5**	**6**	**7**
