# Supplementary material for: Factors Influencing Burnout in Croatian Medical Students: The roles of Lifelong Learning and Loneliness
Source: Perspect Med Educ. 2025 May 13;14(1):274–85. doi: 10.5334/pme.1468 (PMC12082462; doi:10.5334/pme.1468)
Supplement: Supplementary File 4. — Presenting the Spearman correlation coefficients for the MBI-GS according to lifelong learning, point grade average, year of study, and age. [file pme-14-1-1468-s4.pdf]

Supplementary File 4. Spearman correlation coefficients for the Maslach Burnout Inventory scores according to lifelong learning, loneliness, grade point average, year of study, and age

	**Burnout (MBI-GS)**			
	Global score	Exhaustion	Cynicism	Professional efficacy
Lifelong learning (JeffSPLL-MS)	–0.33 ***	–0.21 ***	–0.23 ***	+0.30 ***
Loneliness (SELSA-S)				
Global score	+0.23 ***	+0.14 ***	+0.18 ***	–0.17 ***
Family domain	+0.23 ***	+0.13 ***	+0.21 ***	–0.15 ***
Romantic domain	+0.12 ***	+0.10 ***	+0.08 **	–0.09 **
Social domain	+0.28 ***	+0.15 ***	+0.24 ***	–0.23 ***
Grade point average (GPA)	+0.04	+0.01	+0.13 ***	+0.06
Year of study	+0.06 *	–0.05	+0.23 ***	+0.05
Age	+0.06 *	–0.06 *	+0.22 ***	+0.04

Notes: MBI-GS: Maslach Burnout Inventory–General Survey; JeffSPLL-MS: Jefferson Scale of Physicians Lifelong Learning – Medical student version; SELSA-S: Social and Emotional Loneliness Scale for Adults; *p<0.05; ** p<0.01; ***p<0.001
